# Association of Systemic Pathologies on Dental, Periodontal and Orthodontic Status in Children

**DOI:** 10.3390/biomedicines13092137

**Published:** 2025-09-01

**Authors:** Ioana Monica Teodorescu, Elena Preoteasa, Cristina Teodora Preoteasa, Cătălina Murariu-Măgureanu, Cristian Teodorescu

**Affiliations:** 1 Department of Prosthodontics, Faculty of Dentistry, “Carol Davila” University of Medicine and Pharmacy, 020021 Bucharest, Romania; 2 National Institute for Mother and Child Health “Alessandrescu Rusescu”, 020395 Bucharest, Romania; 3 Department of Scientific Research Methods-Ergonomics, Faculty of Dentistry, “Carol Davila” University of Medicine and Pharmacy, 020021 Bucharest, Romania

**Keywords:** oral pathologies, general pathologies, clinical status, pediatric patients

## Abstract

**Background/Objectives:** This retrospective observational study evaluated associations among dentition type, age, systemic conditions, and oral pathology in pediatric patients. **Methods:** A six-month retrospective analysis was conducted in a specialized pediatric hospital. Patients (*n* = 155; 0–18 years) were grouped as clinically healthy with oro-maxillofacial diseases or with systemic diseases. Data included dental/periodontal status, anomalies, gingival bleeding index, IOTN score and oral mucosal conditions. Statistical tests (chi-square, ANOVA, and correlations) were applied. **Results:** Chronic gingivitis was most frequently associated with primary dentition. Caries and root debris were more frequent in ages 4–6, while acute gingivitis appeared in younger children. Orthodontic treatment need increased with age, especially in mixed dentition. Periodontal diseases were most often linked to digestive pathologies. **Conclusions:** Oral health in children showed associations with age, dentition type, and systemic conditions. Findings highlight associations rather than causation, underscoring the importance of prevention, early monitoring, and age-appropriate interdisciplinary management.

## 1. Introduction

Oral health in children is shaped by caries and periodontal disease, which remain highly prevalent worldwide. While systemic conditions such as digestive, metabolic, and hematological disorders are known to influence oral status in adults, limited data exist on pediatric populations. Prior studies often examine isolated conditions but do not comprehensively compare children with and without systemic disease across dentition stages. This study addresses this gap by examining associations between systemic conditions and dental, periodontal, and orthodontic status in children aged 0–18 years in a hospital-based cohort.

Oral health refers to the condition of the mouth, involving functions such as mastication, speech, and facial movement. It is defined by the lack of maxillofacial diseases and related discomfort and plays a role in overall individual well-being. Dental caries and periodontal diseases represent the most prevalent oral health conditions among pediatric and adolescent populations. According to published research, the prevalence of dental caries is 40%, while gingival pathology affects between 60% and 80% of the population [1].

A study conducted by the American Academy of Pediatric Dentistry (AAPD) indicates that over 2 billion individuals experience dental caries in their permanent teeth, while approximately 520 million children are affected by cavities in their primary teeth. Early childhood caries refers to the presence of carious lesions on one or more surfaces of primary teeth in children up to 71 months old, typically those under six years of age. Despite the AAPD’s consistent emphasis on the importance of pediatric oral health, it is common for young children to seek dental care only when faced with immediate concerns [2].

Dental caries is a prevalent chronic condition in children. If not diagnosed early, more severe complications may arise, necessitating advanced treatments. As a result, prevention, timely diagnosis, and management of dental caries are important for pediatric patients in relation to both oral and overall health [3].

Children with chronic illnesses are more likely to develop oral health problems than their healthy peers. A lack of awareness regarding the connection between general and oral health, reduced motivation, and experiences of discomfort may contribute to difficulties in maintaining oral health for these patients [4].

The deciduous dentition supports mastication, speech, and development of the stomatognathic system. Premature loss, often due to local or systemic factors, can lead to orthodontic needs and psychosocial challenges [5,6].

Recent advances in periodontal health have enhanced our understanding of the pathogenesis and pathophysiology of periodontal diseases, their interactions with the host, and their associations with systemic conditions. While diabetes and cardiovascular disease are most frequently linked to periodontal disease, emerging research indicates that periodontal health may also impact reproductive health and fertility [7].

Vitamin deficiencies may manifest orally as mucositis, enamel defects, petechiae, or ulcers, underlining systemic–oral health links relevant to children [8].

The aim of this study was to investigate the relationship between different general pathologies and their association with oral diseases in individuals 0 to 18 years old.

## 2. Materials and Methods

This was a retrospective observational study conducted over six months (January–June 2024) in a pediatric dental unit. All available patient records (*n* = 155; age 0–18 years) were consecutively reviewed. Patients were classified as (a) clinically healthy with oro-maxillofacial conditions only, or (b) with systemic conditions (acute or chronic). Exclusion criteria included incomplete records and prior orthodontic treatment. Data collected: age, sex, residence, dentition type, dental and periodontal status, IOTN score, gingival bleeding index, and oral mucosal conditions. Diagnostic categories were standardized, and references for indices were added. The gingival bleeding index was simplified (noted as a limitation). No examiner calibration was performed. Statistical analysis used chi-square tests, ANOVA, and correlations, with *p* < 0.05 considered significant. Ethics approval was obtained in 2021 as part of doctoral research; data collection was carried out in 2024.

Some patients received outpatient services, while others were admitted, as inpatients, for various chronic or acute conditions that required dental consultation and treatment.

The records of 155 patients were examined and subsequently categorized into two distinct groups. The first group included patients classified as clinically healthy (*n* = 78) with only oral and maxillofacial diseases. The second group consisted of patients with additional general conditions (*n* = 77), such as congenital hypothyroidism, acute gastroenteritis, urinary tract infections, pharyngitis, allergic rhinitis, diabetes, obesity, bacterial mumps, congenital cataracts, hives, antibiotic or food allergies, thalassemia, sinusitis, anemia, and vitamin D deficiency.

The gingival bleeding index was assessed as follows: a score of 0 represented no gingival bleeding, while a score of 1 indicated the presence of gingival bleeding, either upon palpation or occurring spontaneously. The number of teeth was not considered as a variable in this study because the pediatric participants had between 2 and 20 teeth, and cooperation varied among different age groups. The gingival bleeding score was simplified, which should be acknowledged as a limitation.

The index for oral mucosal diseases was assigned as follows: a coefficient of 0 indicated no diseases, 1 denoted minor ulcers (such as canker sores, herpes, or microtrauma), 2 referred to ulcerative-necrotic gingivostomatitis, 3 signified oral candidiasis, 4 represented oral abscesses, and 8 included other oral cavity diseases. These classifications are based on the World Health Organization’s Children’s Oral Health Assessment Form, 2013 [9].

The Index of Orthodontic Treatment Need (IOTN) was designed to categorize malocclusion based on the necessity for orthodontic intervention and to help determine patient prioritization and is not originally intended for primary dentition, but it was applied across all dentition stages for consistency in comparative analysis. The Dental Health Component (DHC) of the IOTN utilizes a five-level scale that ranks the severity of occlusal characteristics and the corresponding need for treatment: grade 1 indicates no need for orthodontic care, grade 2 denotes a minor need, grade 3 is considered borderline, grade 4 signals a substantial need, and grade 5 represents a very substantial requirement for orthodontic treatment.

DHC-IOTN evaluated malocclusion and dento-maxillary anomalies using five criteria [10]: missing teeth, classified as either less extensive hypodontia (grade 4) or extensive hypodontia with restorative considerations (grade 5); increased overjet (sagittal malocclusion at the incisor level); crossbite; loss of contact points; and deep overbite (vertical malocclusion involving the anterior teeth).

The data was systematically collected and recorded in spreadsheets utilizing Microsoft Office 365 Excel. Subsequently, statistical analyses were conducted with Microsoft Office 365 Excel (Washington, DC, USA) and IBM SPSS Statistics 30 (New York, NY, USA), including calculation of *p*-values, application of the chi-square test and ANOVA, as well as determination of means, standard deviations, and correlation matrices.

The study received approval as part of doctoral research from the pediatric hospital, which supplied statistical data obtained from patient records, with authorization from the Research and Development Ethics Commission of the National Institute for Mother and Child Health “Alessandrescu—Rusescu” in Bucharest (approval number 14222/29.07.2021).

## 3. Results

Among 155 patients, 78 were clinically healthy and 77 had systemic conditions. In healthy children, intact dentition predominated, but one-third had untreated caries and two-thirds had gingival pathology, mainly chronic gingivitis. In children with systemic conditions, caries, root debris, and anterior demineralization were more common, alongside higher prevalence of acute gingivitis and herpetic lesions. Orthodontic anomalies, including class II maxillary compression and mandibular prognathism, were identified more frequently in mixed dentition. Statistical testing showed significant associations between dental and periodontal health in both groups (*p* < 0.05), and between dental and orthodontic status in healthy patients only.

### 3.1. Clinically Healthy Patient Group

Group 1 included 78 clinically healthy children, mostly female, from urban areas, with primary dentition. Over half of them had intact teeth, but a third—mainly females with mixed dentition—showed untreated caries or root debris (Table 1).

Among patients deemed clinically healthy, two-thirds showed gingivo-periodontal issues, mainly chronic gingivitis linked to poor oral hygiene. Other observed conditions included eruption gingivitis, mouth sores, upper lip edema, acute gingivitis, and periodontal abscesses. Gingival bleeding and oral mucosal diseases were rare. Periodontal diseases were most often diagnosed in females with temporary dentition.

About one-third of clinically healthy patients, mostly female, had IOTN scores of 3 or 4, suggesting a borderline or definite need for orthodontic treatment. A quarter of children, particularly girls, presented with class II Angle division 2 maxillary compression. Cases of class II division 1 maxillary compression and one mandibular prognathism were observed among male patients (Table 2).

### 3.2. Group of Patients with Associated General Conditions

The second group consisted of 77 patients (nearly equal numbers of males and females), mostly from urban areas with primary dentition. Over half of them had additional dental issues, notably multiple root debris, simple caries, and maxillary anterior teeth demineralization, primarily in cases of temporary or mixed dentition (Table 3).

Among this patient population, acute gastroenteritis, primary herpes infection, and antibiotic allergies were the most frequently observed general conditions. Additional diagnoses included urinary tract infections, allergic rhinitis, protein-calorie malnutrition, and iron deficiency anemia. Nearly 20% of patients exhibited symptoms localized exclusively to the oral cavity, such as herpangina, primary herpes infection, and bacterial parotitis. Most individuals diagnosed with gastroenteritis were also found to have chronic dental conditions such as caries (both uncomplicated and complicated), multiple root debris, or demineralization of the maxillary anterior teeth.

Many patients with general comorbidities presented with periodontal conditions, with chronic gingivitis being the most diagnosed, followed in prevalence by acute gingivitis, eruption gingivitis, and acute herpetic gingivitis. Additionally, a single case of Bohn’s nodules or an epulis-type oral tumor was observed. Temporary dentition was primarily affected, although eruption gingivitis was also present in patients with mixed dentition. Approximately one-fourth of the patients exhibited positive bleeding index or alterations in the oral mucosa. These rare findings should be interpreted cautiously given the very small numbers. The orthodontic assessment of patients in the second group revealed moderate incidences of class II Angle division 2 maxillary compression, mandibular prognathism, and edge to edge occlusion, with these conditions observed more frequently during the mixed dentition period. Based on the IOTN score, approximately 20% of patients exhibited some degree of orthodontic treatment need, ranging from low to very high (Table 4).

### 3.3. Comparative Statistical Evaluation of Pediatric Patient Cohorts

A comparative analysis of *p*-values for correlations among dental, periodontal and orthodontic statuses in the two patient groups revealed a statistically significant association between dental and periodontal health (*p* < 0.05) in both cohorts. Among clinically healthy individuals, dental health was found to influence the need for orthodontic intervention (*p* < 0.05); however, this relationship was not observed in patients with comorbid conditions. Additionally, no association between periodontal status and dento-maxillary anomalies was identified in either group.

For the clinically healthy patient group, Chi-square tests showed a statistically significant association between dental and gum health, particularly among female patients. A relationship between dental health and orthodontic needs was also observed, with this effect being more notable in male patients. Anova tests indicated that age has a slight influence on periodontal status, although the relationship is weak. Significant differences were identified between age categories regarding dento-maxillary anomalies. There was a correlation between age, bleeding index, and IOTN score: younger patients tend to have higher bleeding indices, which is associated with greater susceptibility to periodontal disease; older patients are more likely to have higher IOTN scores. The correlation between mucosal disorders and orthodontic treatment need was not statistically significant (Table 5).

Among patients with associated conditions, Chi-square tests identified correlations between certain diagnoses and periodontal health, specifying patterns related to gum issues. A moderate association was noted between age and general diagnoses, reflecting specific trends for some age-related conditions. The data also indicated that age affects periodontal health, with variations observed across different age groups in the studied population. Furthermore, orthodontic needs were found to be influenced by age, consistent with developmental patterns (Table 6).

### 3.4. Age-Related Trends in Dental, Periodontal and Orthodontic Status

In clinically healthy patients, trends indicate that dental status has remained largely unchanged in younger children aged 0 to 5 years, consistent with the less complex dental requirements of primary teeth. In contrast, older children between 6 and 15 years tend to experience an increase in dental issues and dento-maxillary anomalies, emphasizing the relevance of regular check-ups for early detection and management of dental conditions.

The periodontal status of this group of patients showed that gingival health remains largely consistent across most ages, with some variations observed in certain age ranges, which may correspond to phases such as tooth eruption or gingivitis. Studies on dento-maxillary anomalies demonstrate that orthodontic concerns are relatively rare in young individuals with primary dentition but tend to increase in prevalence with age, as alterations in tooth positioning or occlusion become more common.

**Figure 1:**
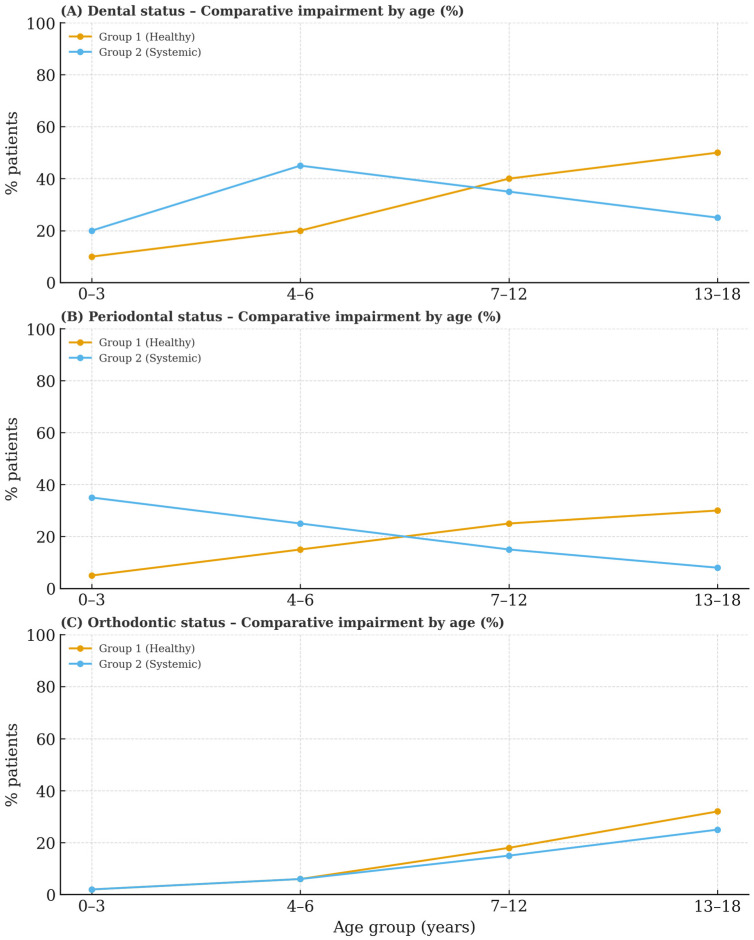
Comparative status impairment percentage by age.

In patients with associated general conditions, analysis of age-related trends in dental, periodontal and orthodontic statuses indicates that dental diseases are most prevalent among individuals aged 4 to 6 years, with frequency decreasing as age increases. Periodontal problems are most common in the 0–3-year age group and show notable improvement by ages 13–18. Dento-maxillary anomalies are infrequent in younger patients but become more common during adolescence (ages 13–18), aligning with an increased demand for orthodontic treatments (Figure 1).

The statistics show patterns that can help predict changes in the oral health of children and adolescents. The average age of patients with demineralization of the maxillary anterior teeth was 2.5 years, which is commonly observed in younger individuals. In contrast, the average age was 6.29 years for those with multiple simple caries and 6.19 years for those with multiple root debris, suggesting that these dental issues are more common in slightly older children. The “healthy” group, which refers to patients without significant dental problems, had the highest standard deviation (4.5), indicating a broader age distribution within this category. Severe dental conditions, including “multiple untreated simple and complicated caries”, exhibited lower variability, implying they occur within a more restricted age range.

Regarding periodontal status, “chronic gingivitis” was more commonly observed in children with an average age of 6.77 years, compared to “eruption gingivitis” (mean age 4.83 years) and “acute herpetic gingivitis” (mean age 1.75 years), indicating a tendency for chronic gingivitis to develop over time. Patients “without periodontal issues” had a mean age of 4.5 years, closely aligning with the gingivitis cases. The largest standard deviation was noted among cases of “acute gingivitis” (7.09 years), suggesting its occurrence across a broad age range, whereas “acute herpetic gingivitis” appeared within a more restricted age group. Notably, both acute and chronic gingivitis presented with wider age ranges, demonstrating that these conditions can affect children at various developmental stages.

**Figure 2:**
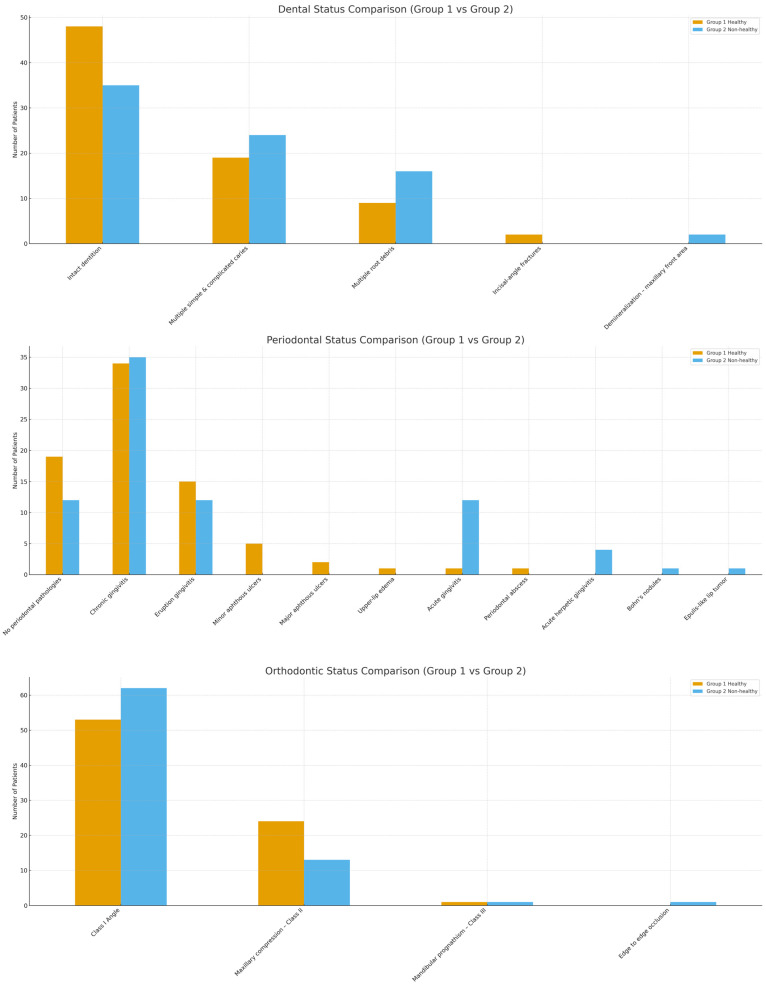
Comparative diagnostics between group 1 and 2.

Figure 2 presents diagnostic frequencies for the two groups. Children with systemic conditions had higher rates of untreated caries, root debris, and anterior demineralization than those without systemic conditions. Greater prevalence of periodontal pathologies, including chronic and acute gingivitis, was identified in the systemic group. Orthodontic anomalies such as maxillary compression and prognathism were reported at similar frequencies in both groups, with no statistically significant difference (χ^2^, *p* > 0.05). Statistically significant differences were noted in dental and periodontal status (*p* < 0.05), indicating an association between systemic conditions and oral disease burden. Comparative analysis shows that the presence of systemic conditions is associated with increased dental and periodontal complications, while observed orthodontic patterns are consistent across both groups. These results suggest that systemic disease may influence inflammatory and carious processes but does not significantly affect occlusal development.

## 4. Discussion

The relationship between oral health and systemic diseases is a subject of significant interest for general practitioners, dental professionals, and researchers, as demonstrated by its frequent discussion in published literature [7]. Digestive disorders, as well as metabolic, hematological, and endocrine conditions, are commonly linked to oral pathologies [11], as indicated by the findings of this study. Jajam M, Bozzolo P and Niklander S [12] have also highlighted the oral manifestations that pathologies in the digestive sphere such as Crohn’s disease, ulcerative colitis, celiac disease or gastroesophageal reflux can have on oral tissues, manifestations that often precede systemic symptoms [13,14,15]. Periodontal and gingival diseases are frequently observed in these contexts, including conditions such as oral ulcers, lithiasis, mucogingivitis, labial and facial inflammation, pyostomatitis, and dysgeusia [12], aspects also highlighted in our study.

The second most frequently observed general pathology was primary herpetic infection, a condition with recognized oral manifestations as documented in the literature [16]. Ortis M et al. [17] examined the effects of herpes simplex virus type 1 (HSV-1) on oral epithelium, connective tissue, and neurons, and reported that the periodontal ligament may serve as a reservoir for the virus, potentially contributing to the progression of periodontal diseases. These lesions are most commonly found in the gingival mucosa, vermilion, and tongue, as has been documented by Țovaru Ș et al. [18], and their study observed that gingivitis and pharyngo-tonsillitis associated with primary herpes infection occurred more frequently among pediatric patients. Specifically, acute herpetic gingivitis was identified in four children within our study who also presented with underlying health conditions; notably, oral mucosal changes were observed in one quarter of this patient group. In fact, the primary herpes infection can also mimic other viral or bacterial infections, Huang CW et al. [19] describing symptoms that include fever, exudate-covered tonsils, oral ulcers, inflammation, and bleeding gums.

An important category of pediatric patients with oral diseases have been diagnosed with hypovitaminosis, Bačun B et al. [20] highlighting the frequent correlations between these two pathologies. Deficiencies in vitamins B, E, or D were often associated with lesions of the oral mucosa, such as canker sores or ulcers. Vitamin C and D deficiencies have been associated with periodontal diseases characterized by inflammation and gingival bleeding. This study found that approximately two-thirds of children classified as clinically healthy exhibited periodontal disease, with similar findings observed in pediatric patients who had additional comorbidities; gingivitis and canker sores were the most frequently observed conditions. Lešić S et al. [8] examined how vitamins affect oral health, finding that deficiencies in vitamins A, K, and D are linked to dental lesions like enamel hypo mineralization, while lack of B or C vitamins often results in gum, tongue, or mucosal diseases. Our study also observed demineralization of upper front teeth and caries in children with systemic conditions. The primary factor contributing to the rising prevalence of periodontal diseases among children was inadequate oral hygiene. Olczak-Kowalczyk D et al. [21] also indicated that socioeconomic factors and dietary habits may influence the onset and progression of gingivitis, which is also affected by dental status. Moreover, our study revealed a significant correlation between dental and periodontal health in pediatric patients, both among those deemed clinically healthy and those presenting with associated systemic conditions.

Regarding the evolution of dental pathologies according to age, Han SY et al. [22] identified that the prevalence of oral diseases varies by age group: dental diseases are most common among children aged 0–3 years; dento-maxillary disharmonies predominantly affect preschoolers aged 4–6 years; and periodontal problems are most frequently observed in adolescents over 13 years old. Our research shows associations with early diagnosis of dental conditions and disharmonies, particularly from age six onward, coinciding with the onset of permanent tooth eruption. Nonetheless, we observed variations in age-related patterns when comparing clinically healthy children to those presenting with comorbid general conditions. Consequently, for children within the first category, dental diseases are infrequent during early childhood. However, as mixed dentition develops, there is an increased exposure to risk factors—including diet and suboptimal oral hygiene—as well as notable changes in dental, periodontal and orthodontic status. Furthermore, a statistically significant correlation exists between dental and periodontal statuses, as well as between dental status and dento-maxillary anomalies. Conversely, among pediatric patients with systemic diseases, distinct dental issues were observed across age groups: periodontal conditions were noted in children up to 3 years old during the eruption of primary teeth; dental concerns predominated in those undergoing mixed dentition; and dento-maxillary anomalies were most prevalent in adolescents aged 13 to 18 years.

Among the most common general pathologies, allergic rhinitis are correlated with dento-maxillary disharmonies, and Farronato M et al. [23] noted that mouth breathing and nasal obstruction are specific features of allergic rhinitis, and children exhibiting these symptoms have a higher prevalence of malocclusions.

The impact on oral microbiota should not be overlooked; in this respect, Contaldo M et al. [24] highlighted the quantitative and qualitative changes that the oral microbiota can undergo in patients with orthodontic treatments, in whom the predisposition for caries or periodontal diseases is higher, with an impact on general health. At the same time, there is an interrelationship between malocclusion, head position and body posture [25], and a multidisciplinary approach to this category of pediatric patients is needed.

Congenital hypothyroidism is a frequently occurring endocrine disorder in pediatric patients and was also observed in the second group of our study. Numerous studies have identified oral manifestations of this pathology, such as enamel hypoplasia [26], glossitis, open bite and dental crowding [27], delays in tooth eruption, anodontia, ectopia or tooth decay [28], and indeed, dental caries—whether simple or complex—represented the most prevalent dental condition observed within this patient group.

A subset of patients experienced allergic reactions to antibiotics or foods, resulting in oral manifestations including cheilitis, aphthous stomatitis, and erythema. These clinical signs have also been documented by Dayanarayana U et al. [29], who observed that these manifestations may resemble those of other common diseases, potentially complicating the process of establishing a differential diagnosis. Within this context, Toader SV et al. [30] advise conducting skin patch tests and performing histopathological evaluations to accurately identify the underlying cause of allergic-type reactions. A distinct subset of these conditions involves immediate or delayed responses to the materials utilized in orthodontic appliances. The scientific literature reports the development of ulcerative-erosive lesions and gingival hyperplasia as potential outcomes [31]. Adolescent patients, who often exhibit a greater need for orthodontic intervention, should be systematically and carefully monitored in this context.

This study found significant associations between systemic pathologies and oral conditions in children. Digestive disorders were most strongly linked with periodontal disease, consistent with prior reports of gastrointestinal related oral manifestations. Vitamin deficiencies correlated with enamel defects, caries, and periodontal inflammation, echoing findings by Lešić et al. Allergic rhinitis was associated with dento-maxillary disharmonies, likely mediated by mouth breathing, as shown in previous reviews. In contrast to much of the literature, younger children in our cohort showed higher bleeding indices, likely reflecting sample bias and acute conditions in early dentition. Rare conditions (e.g., herpetic gingivitis) were observed but should be interpreted cautiously due to the small numbers. Strengths of the study include the inclusion of both healthy and systemically affected children, and assessment across dentition stages.

Limitations include retrospective design, hospital-based sample, small subgroups, the lack of examiner calibration, and unmeasured confounders (diet, hygiene, and socioeconomic factors), the use of IOTN in primary dentition, and the uneven distribution of dentition types. Future prospective community-based studies are needed to validate these associations and assess clinical significance. Our findings suggest pediatric dental monitoring should integrate systemic health screening for improved preventive care.

## 5. Conclusions

This retrospective study identified associations between systemic and oral pathologies in pediatric patients. Periodontal diseases were often linked to digestive conditions, while dental status correlated with orthodontic anomalies in healthy children. No causal inference can be drawn due to the study design, but findings emphasize the need for early preventive care, systemic disease management, and interdisciplinary pediatric monitoring. This study establishes a foundation for future research, which could be enhanced by utilizing larger, community-based samples and standardized assessment tools.

## Figures and Tables

**Table 1:** Numerical and percentage distribution of patients in group 1 according to sex, environment of origin and type of dentition.

Group	No. of Patients	Girls	Boys	Urban Environment	Rural Environment	Primary Dentition	Mixed Dentition	Permanent Dentition
**1**	**78**	45 (57.69%)	33 (42.31%)	63 (80.77%)	15 (19.23%)	43 (56%)	28 (35%)	7 (9%)

**Table 2:** Numerical and percentage distribution of patients in group 1 according to dental, periodontal and orthodontic status.

	Number	Percentage
**DENTAL STATUS**		
**Intact dentition**	48	61.54
**Untreated multiple simple and complicated caries**	11	14.1
**Multiple root debris**	9	11.54
**Multiple simple caries**	5	6.41
**Incisal-angle fractures (trauma)**	2	2.56
**Partially treated multiple caries**	2	2.56
**Treated multiple caries**	1	1.28
**PERIODONTAL STATUS**		
**No periodontal pathologies**	19	24.36
**Chronic gingivitis**	34	43.59
**Eruption gingivitis**	15	19.23
**Minor aphthous ulcers**	5	6.41
**Major aphthous ulcers**	2	2.56
**Upper-lip edema**	1	1.28
**Acute gingivitis**	1	1.28
**Periodontal abscess**	1	1.28
**ORTHODONTIC STATUS**		
**Class I Angle**	53	67.94
**Maxillary compression—Class II Div. 2 Angle**	21	26.92
**Maxillary compression—Class II Div. 1 Angle**	3	3.84
**Mandibular prognathism—Class III Angle**	1	1.28

**Table 3:** Numerical and percentage distribution of patients in group 2 according to sex, environment of origin and type of dentition.

Group	No. of Patients	Girls	Boys	Urban Environment	Rural Environment	Primary Dentition	Mixed Dentition	Permanent Dentition
**2**	**77**	39 (50.65%)	38 (49.35%)	48 (62.34%)	29 (37.66%)	48 (62.34%)	23 (29.87%)	6 (7.79%)

**Table 4:** Numerical and percentage distribution of patients in group 2 according to dental, periodontal and orthodontic status.

	Number	Percentage
**DENTAL STATUS**		
**Intact dentition**	35	45.45
**Multiple root debris**	16	20.78
**Multiple simple and complicated caries**	12	15.58
**Simple caries**	12	15.58
**Demineralization—maxillary front area**	2	2.6
**PERIODONTAL STATUS**		
**No periodontal pathologies**	12	15.58
**Chronic gingivitis**	35	45.45
**Acute gingivitis**	12	15.58
**Eruption gingivitis**	12	15.58
**Acute herpetic gingivitis**	4	5.19
**Bohn’s nodules**	1	1.29
**Epulis-like lip tumor**	1	1.29
**ORTHODONTIC STATUS**		
**Class I Angle**	62	80.51
**Maxillary compression—Class II Angle**	13	16.88
**Mandibular prognathism Class III Angle**	1	1.29
**Edge to edge occlusion**	1	1.29

**Table 5:** Statistical analysis of patients in group 1.

Comparison (Status)	Chi-Square (χ^2^)	*p*-Value
Dental vs. Periodontal	68.55	0.00597
Dental vs. Orthodontic	86.95	5.08 × 10^−11^
Periodontal vs. Orthodontic	17.66	0.670

**Table 6:** Statistical analysis of patients in group 2.

Comparison (Status)	Chi-Square (χ^2^)	*p*-Value
Dental vs. Periodontal	60.91	4.74 × 10^−5^
Dental vs. Orthodontic	11.06	0.524
Periodontal vs. Orthodontic	16.68	0.545

## Data Availability

The data presented in this study are available on request from the corresponding authors.

## Abbreviations

The following abbreviations are used in this manuscript:

IOTN	Index of Orthodontic Treatment Need
AAPD	American Academy of Pediatric Dentistry
ECC	Early childhood caries
